# Hematuria and Proteinuria in a Patient with Monoclonal Gammopathy of Renal Significance

**DOI:** 10.34067/KID.0000000957

**Published:** 2026-02-26

**Authors:** Muying Zhang, Ying Fu, Xiao-jun Chen

**Affiliations:** Department of Nephrology, Second Xiangya Hospital, Central South University, and Hunan Provincial Key Laboratory of Kidney Disease and Blood Purification, Changsha, China

**Keywords:** albuminuria, clinical nephrology, glomerular disease, kidney biopsy

## Abstract

**Figure:**
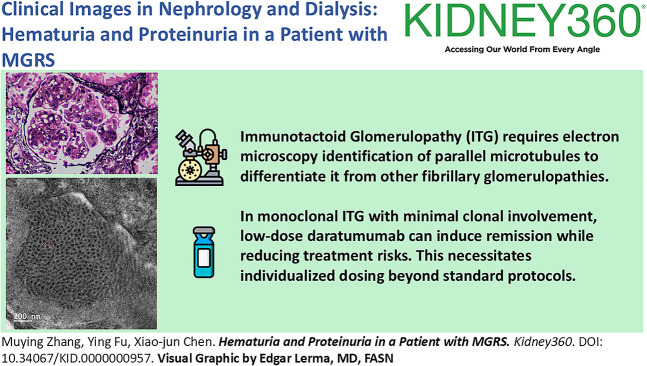

## Case Description

A 72-year-old male presented with bilateral lower limb edema and fatigue accompanied by hypertension (150/80 mm Hg). Laboratory investigations revealed significant proteinuria of 3.4 g/gCr, hematuria (red blood cells 40.0/*μ*l, 70% dysmorphic), serum creatinine of 1.1 mg/dl, and eGFR of 62.4 ml/min per 1.73 m^2^. Further quantification demonstrated 24-hour urine protein excretion of 4.3 g/day with severe hypoalbuminemia (serum albumin 2.1 g/dl). Serum and urine protein electrophoresis/immunofixation identified IgGκ-type monoclonal protein. Free light chain κ was 70.5 mg/L, and free light chain λ was 43.7 mg/L. Tests for hepatitis B and C viruses, antinuclear antibodies, antineutrophil cytoplasmic antibodies, and double-stranded DNA antibodies were negative. Cryoglobulin and malignancy screenings were all negative. Flow cytometric analysis of the bone marrow revealed 0.4% abnormal monoclonal plasma cells.

**Figure 1:**
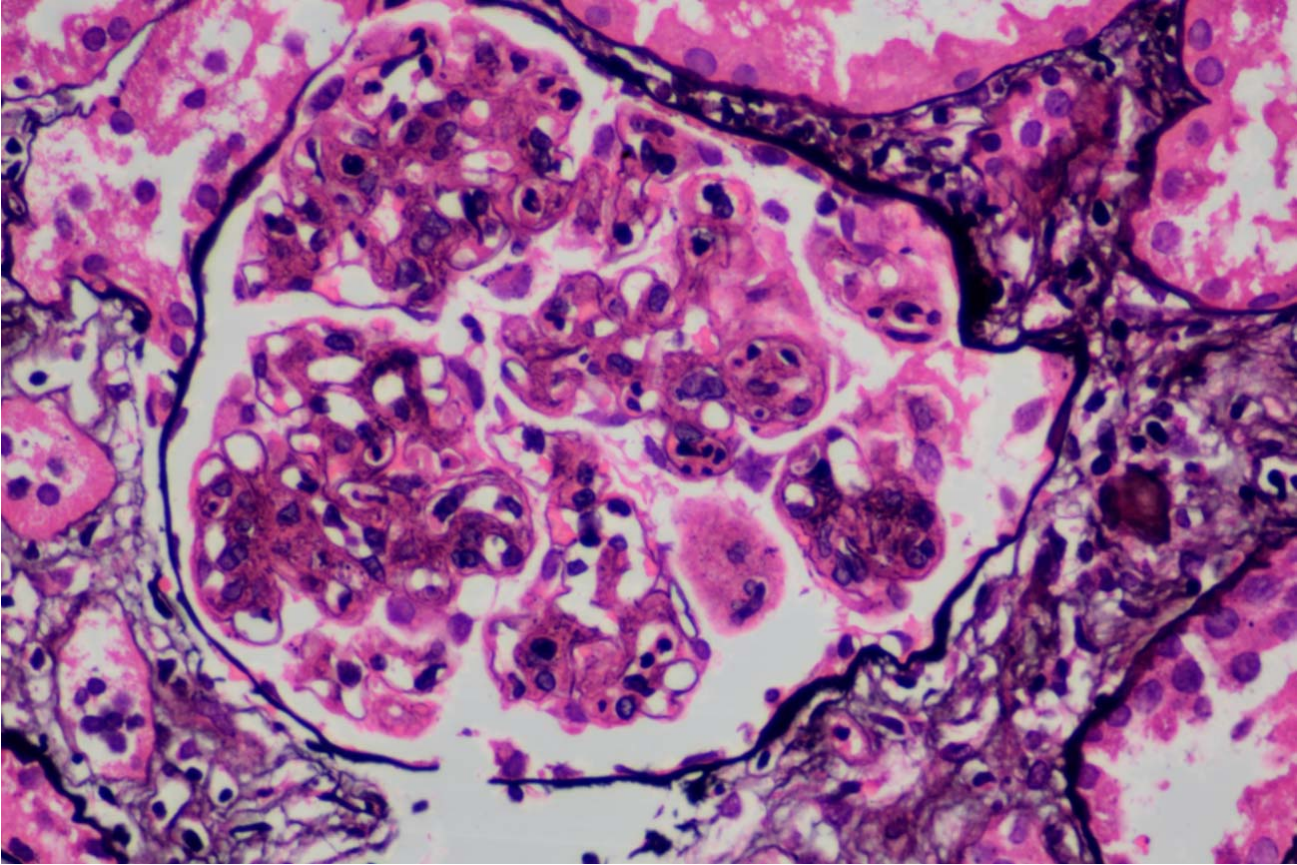
**Light microscopy findings in ITG.** Light microscopy revealed mesangial hypercellularity, matrix expansion, capillary loop lobulation, endothelial cell swelling, and basement membrane thickening with spike formation (PAS methenamine staining, original magnification ×400). ITG, immunotactoid glomerulopathy; PAS, Periodic Acid–Schiff.

**Figure 2:**
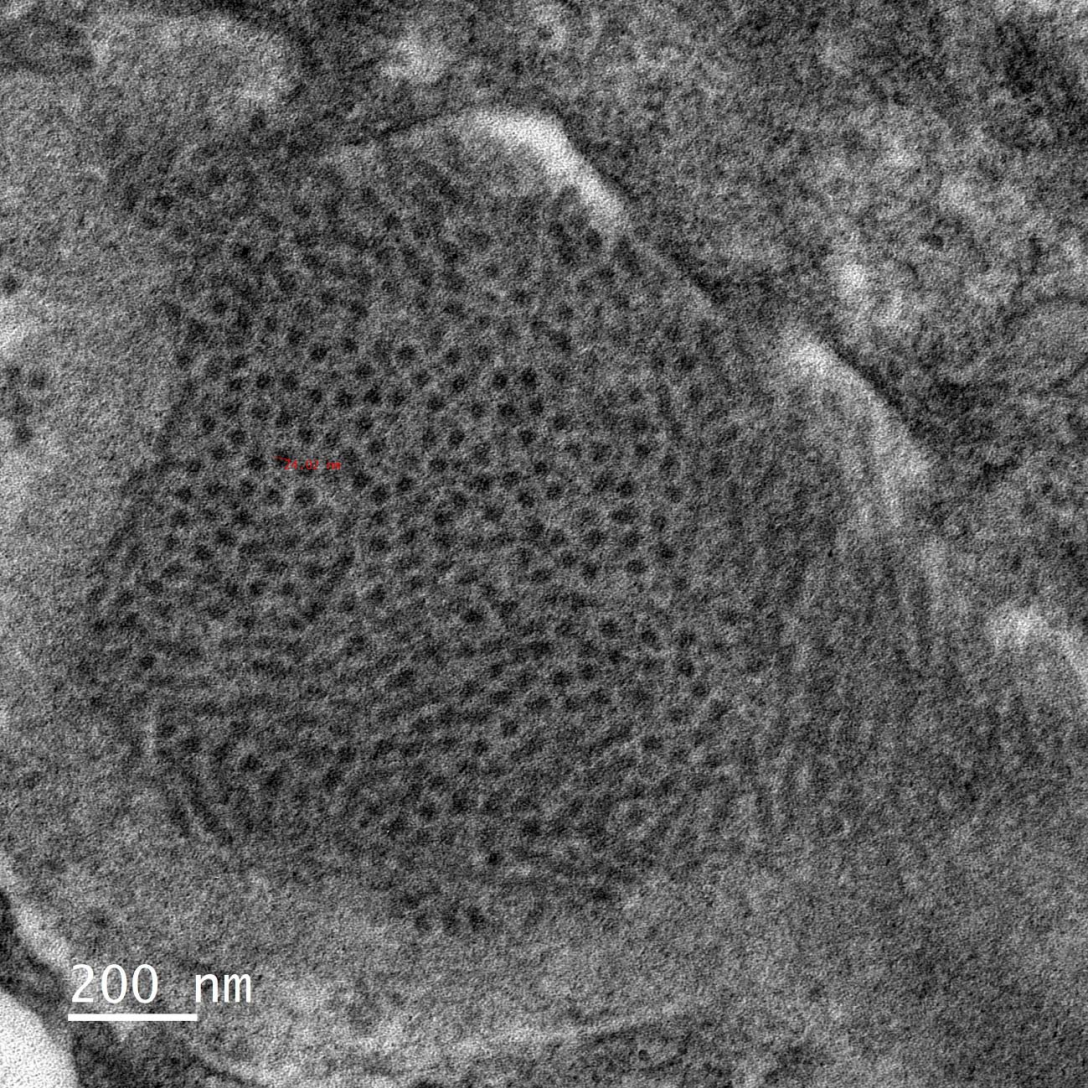
**Electron microscopy findings in ITG.** On electron microscopy, the deposits were composed of microtubules with a diameter of almost 25 nm and a distinct hollow core (Original magnification ×12,000).

Kidney biopsy immunofluorescence exhibited prominent IgG (+++) and C3 (+++) deposition in glomeruli, with focal IgM (+/−) and C1q (+/−). Kappa light chains were strongly positive, while lambda staining was negative. Light microscopy revealed mesangial hypercellularity, matrix expansion, capillary loop lobulation, endothelial cell swelling, and basement membrane thickening with spike formation. Mesangial interposition and double contours were observed (Figure 1). Electron microscopy revealed microtubules with a diameter of approximately 25 nm and a distinct hollow core, which were arranged in parallel alignment (Figure 2). These findings confirmed the diagnosis of immunotactoid glomerulopathy (ITG) associated with monoclonal gammopathy.

Initial therapy comprised dexamethasone (10 mg every 2 weeks), bortezomib (1.3 mg/m^2^ every 2 weeks), and tacrolimus (0.5 mg twice daily). After 3 months, despite incomplete resolution of edema, proteinuria progression was evidenced by an increased urine protein-to-creatinine ratio. Continuation of this regimen through October 2024 yielded no significant improvement. Following treatment discontinuation in December 2024, clinical deterioration occurred by January 2025. Therapy was subsequently modified to daratumumab (100 mg every 2 months) combined with tacrolimus (0.5 mg twice daily). After the initial daratumumab dose, serum free κ and λ light chains decreased precipitously. Over the ensuing 6 months, bilateral edema resolved completely, serum albumin rose to 3.9 g/dl, and the urine protein-to-creatinine ratio returned to 1.8 g/gCr. Serum free light chains remained at low levels throughout follow-up.

## Discussion

ITG, a rare chronic glomerulopathy encountered in 0.06% of native kidney biopsies,^1^ is ultrastructurally defined by microtubular deposits and currently lacks standardized therapeutic protocols. Existing clone-directed approaches, such as rituximab, alkylating agents, and bortezomib, have demonstrated suboptimal efficacy.^2,3^ The pathogenesis of monoclonal ITG remains elusive; however, its frequent hematological associations^4^ imply that targeting underlying clonal disorders may enhance renal outcomes. Treatment strategies prioritize achieving sustained hematological remission through clonally targeted therapy, which involves B-cell–directed agents for lymphoproliferative clones and plasma cell–targeted regimens for plasmacytic clones.^2^ We first reported that low-dose daratumumab was associated with significant clinical and biochemical remission in monoclonal ITG, highlighting its potential as a promising therapeutic option.

## Teaching Points

- ITG requires electron microscopy identification of parallel microtubules to differentiate it from other fibrillary glomerulopathies.
- In monoclonal ITG with minimal clonal involvement, low-dose daratumumab can induce remission while reducing treatment risks. This necessitates individualized dosing beyond standard protocols.
